# Synergistic Effects of Time-Restricted Feeding and Resistance Training on Body Composition and Metabolic Health: A Systematic Review and Meta-Analysis

**DOI:** 10.3390/nu16183066

**Published:** 2024-09-11

**Authors:** Yiling Ho, Xiao Hou, Fenghua Sun, Stephen H. S. Wong, Xiaoyuan Zhang

**Affiliations:** 1 Department of Physical Education, Peking University, Beijing 100871, China; 2201212188@stu.pku.edu.cn; 2 School of Sport Science, Beijing Sport University, Beijing 100871, China; houxiao0327@bsu.edu.cn; 3 Department of Health and Physical Education, The Education University of Hong Kong, Hong Kong, China; fhsun@eduhk.hk; 4 Department of Sports Science and Physical Education, Faculty of Education, The Chinese University of Hong Kong, Hong Kong, China; hsswong@cuhk.edu.hk

**Keywords:** time-restricted feeding, resistance training, body composition, metabolic health

## Abstract

Background: This systematic review and meta-analysis examined the synergistic impact of time-restricted feeding (TRF) combined with resistance training (RT) (TRF + RT) on body composition and metabolic health in adults, contrasting it with habitual eating patterns (CON) and RT (CON + RT). Methods: Adhering to PRISMA guidelines, five databases were searched up to 28 April 2024. Randomized controlled trials or crossover trials assessing the effects of TRF + RT for at least 4 weeks in adults were selected. Data were pooled as standardized mean differences (SMDs) or weighted mean differences (WMDs) with 95% confidence intervals (CIs). The risk of bias was evaluated using the revised Cochrane risk-of-bias tool. Results: Seven studies with 164 participants were included in the final analysis. TRF + RT significantly reduced body mass (WMD −2.90, 95% CI: −5.30 to −0.51), fat mass (WMD −1.52, 95% CI: −2.30 to −0.75), insulin (SMD −0.72, 95% CI: −1.24 to −0.21), total cholesterol (WMD −9.44, 95% CI: −13.62 to −5.27), low-density lipoprotein cholesterol (LDL-C) (WMD −9.94, 95% CI: −13.47 to −6.41), and energy intake (WMD −174.88, 95% CI: −283.79 to −65.97) compared to CON + RT. No significant changes were observed in muscle mass, strength, or other metabolic markers. Conclusions: TRF + RT, in contrast to CON + RT, significantly improved body composition, insulin, and cholesterol levels without affecting muscle mass or strength.

## 1. Introduction

The global-scale focus on health and physical fitness has intensified with our increasing understanding of their crucial roles in preventing disease, enhancing quality of life, and increasing longevity [1,2]. Dietary strategies and exercise regimens are central to these goals. However, the key to optimizing health outcomes lies not in adopting these strategies individually but rather in recognizing their synergistic potential when judiciously combined [3].

Time-restricted feeding (TRF), a contemporary dietary strategy, restricts food intake to specific time windows, typically extending overnight fasting while allowing ad libitum energy intake during a confined feeding window [4]. TRF has roots that echo ancient religious practices such as those observed during Ramadan [5]. However, it differs from these traditions and other dietary strategies, such as caloric restriction (CR) [6], primarily through its focus on the timing of food intake rather than on the quantity of calories consumed [7]. This approach offers a unique advantage in that it does not necessarily require a reduction in calorie intake; rather, TRF permits periods of standard or increased caloric consumption [8]. This flexibility can lead to better adherence in contemporary populations [9]. Moreover, TRF has the potential to improve metabolic markers, reflecting better glycemic control, improved lipid profiles, and regulated blood pressure [10]. These positive effects can be attributed to changes in metabolic pathways, such as enhanced lipolysis and autophagy [11].

In comparison, resistance training (RT) primarily enhances physical fitness by increasing muscle mass and strength [12]. A common challenge in body weight management strategies, including TRF [13,14] and intermittent fasting [15], is the potential for muscle loss. Maintaining muscle mass is crucial not only for overall health but also for effective body weight control, as it helps sustain the basal metabolic rate [16]. RT plays a vital role in preserving muscle mass or fat-free mass (FFM), thereby aiding in the regulation of body weight [17]. The combination of dietary strategies such as TRF with RT may not only counteract the muscle loss often associated with caloric restriction but also optimize body composition and metabolic health [18,19].

A recently published meta-analysis [20] supported the effectiveness of combining TRF with exercise in reducing body weight and fat mass (FM) as well as improving lipid profiles. However, further research is needed to investigate the effects of different exercise modalities, such as RT. Emerging literature suggests a promising interplay between dietary strategies and RT, implying a synergistic effect that surpasses their individual contributions [21]. RT usually involves a variety of exercises targeting different muscle groups to promote overall muscle growth and strength enhancement, such as bench press, leg press, leg extension, and so on. Preliminary evidence indicates that muscle mass and strength are preserved when these strategies are integrated, even with reduced energy intake [22]. However, whether the effects of TRF combined with RT are comparable to or exceed those of habitual dietary strategies combined with RT remains unknown. Addressing these issues is pivotal for guiding optimal health strategies for the general population seeking health and for individuals like athletes aiming for peak performance.

Therefore, the primary objective of this systematic review and meta-analysis is to address the significant knowledge gap on the impact of combined TRF and RT on health outcomes. We hypothesized that the integrated application of TRF and RT will not only enhance body composition and metabolic health but also potentially mitigate muscle mass loss, a common consequence of weight reduction interventions. Our aim was to elucidate the distinct advantages and limitations inherent in the TRF + RT approach, setting it against the conventional RT with habitual meal patterns (CON + RT). By synthesizing evidence from these comparisons, we intend to offer a well-substantiated perspective, thereby equipping practitioners, athletes, and the wider public with the knowledge to navigate their health decisions.

## 2. Materials and Methods

### 2.1. Protocol and Registration

This systematic review and meta-analysis followed the guidelines outlined in the Preferred Reporting Items for Systematic Reviews and Meta-Analyses (PRISMA). The review protocol was registered on PROSPERO (http://www.crd.york.ac.uk/PROSPERO) (accessed on 25 May 2023) with the registration number CRD42023417177.

### 2.2. Search Strategies

The following databases were searched through 7 April 2023, and updated on 28 April 2024: MEDLINE, Cochrane Library, Embase, CINAHL, and SCOPUS. The search strategy included terms related to the dietary intervention for TRF and RT protocol. The following search terms were used in MEDLINE: tiab ((time restricted feed*) OR (time restricted eat*) OR (time restricted fast*) OR (time restricted diet*) OR (time restricted meal) OR (TRF)) AND tiab ((resistance train) OR (resistance exercis*) OR (weight lift*) OR (weightlift*) OR (weight train*) OR (strength exercis*) OR (strength train*) OR (strengthen*) OR (resistive exercis*) OR (resistive train*)).

### 2.3. Eligibility Criteria

Only randomized trials that included both parallel and crossover designs and met the following criteria were selected: (i) participants aged 18 years or older, (ii) minimum duration of 4 weeks, (iii) implementation of TRF as the dietary intervention, (iv) inclusion of detailed descriptions of the RT protocol, (v) examination of the effects of TRF + RT compared to those of CON + RT, and (vi) evaluation of outcomes related to body composition outcomes (i.e., body mass (BM), FFM, FM, muscle mass, or muscle volume). Research involving individuals with certain medical conditions, individuals taking medications, or those with diseases/conditions known to influence metabolism/body composition were excluded. Review articles, case studies, abstracts, protocols, and conference papers were also excluded.

### 2.4. Data Extraction

During the literature review, two researchers (Y.H. and X.Z.) independently screened the identified titles and abstracts, followed by a full-text review using the earlier eligibility criteria. Duplicate entries were eliminated. Discrepancies were resolved through discussions with a third reviewer (X.H.). These researchers also conducted a detailed review of relevant studies. A specialized form was designed to collect specific information, including the authors’ names, year of publication, sample size, participants’ demographics, study framework, intervention details (covering methods, protocols, and duration), and critical findings. Endnote 20.5 for MAC was used to organize the references. As the primary author, Y.H. oversaw the data collection process, and discrepancies were addressed and resolved collaboratively.

### 2.5. Data Synthesis

For each study, the results were summarized by (i) intervention characteristics (dietary strategies, exercise regimens, and duration), (ii) changes in BM, (iii) changes in FFM, (iv) changes in FM, (v) changes in muscle morphology, (vi) changes in muscle performance, and (vii) changes in blood biomarkers.

### 2.6. Data Analysis

Analyses were conducted using RStudio software version 2023.06.1 + 524. Separate meta-analyses were performed for each outcome (BM, FFM, FM, muscle morphology, muscle strength, energy intake, and blood biomarkers). The meta-analyses were conducted using both random- and fixed-effect models, in which the summary effect size (ES) was the standardized mean difference (SMD) or the weighted mean difference (WMD) of the distribution. In our meta-analysis, the selection between fixed- and random-effects models was based on the observed heterogeneity among studies. A fixed-effect model was chosen for low heterogeneity (*I*^2^ < 40%), assuming a single underlying effect size across the studies. For high heterogeneity (*I*^2^ > 40%), indicating variations in effect sizes, a random-effects model was preferred to account for both within- and between-study variability. This decision was guided by heterogeneity statistics such as the *I*^2^ statistic and Q test. A sequence of sensitivity analyses was conducted by omitting one study at a time.

### 2.7. Risk of Bias

The revised Cochrane risk-of-bias tool for randomized trials (RoB 2) [23] was used to assess the risk of bias. Five domains of bias were identified: (1) randomization process, (2) deviations from the intended interventions, (3) missing outcome data, (4) measurement of the outcome, and (5) selection of the reported result. Overall bias for each item was rated using the following rating categories: (1) low risk of bias, (2) some concerns of bias, and (3) high risk of bias. All selected studies were independently scored by two authors (Y.H. and X.Z.). Any disagreements were resolved by discussion; a third assessor (X.H.) was consulted in case of a disagreement.

### 2.8. Publication Bias

When a meta-analysis encompassed at least six studies, the assessment for publication bias was conducted using a funnel plot and Egger’s test. The analyses were performed utilizing the ‘metal’ package within Stata software, version 18.0 (StataCorp, College Station, TX, USA).

## 3. Results

### 3.1. Study Inclusion

**Figure 1:**
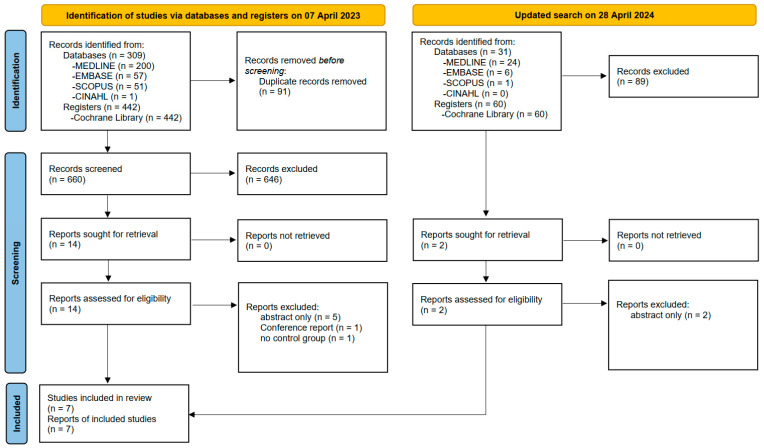
PRISMA flow diagram of study selection.

The systematic search identified 309 studies from databases and 442 studies from registers. After removing duplicates, 660 titles and abstracts were reviewed. Full-text articles were obtained for 14 studies, and their eligibility for inclusion was assessed. Among these, one conference report, five abstracts, and one study lacking a control group were excluded. The remaining seven studies involving 164 participants reported adequate outcome data and were included in this systematic review and meta-analysis. An updated search on 28 April 2024 identified 91 studies in total, and no study was included finally (Figure 1).

### 3.2. Study Characteristics

The characteristics of all the included studies are summarized in Table 1. Seven studies were included in this meta-analysis. Among the total sample size of 164 participants, 83 were in the TRF + RT group, and 81 were in the CON + RT group. Most studies focused on active individuals, with only one study targeting overweight and obese adults. The average age of the participants ranged from 22.3 to 45 years, and the BM index (BMI) ranged from 22.5 to 29.8 kg/m^2^. The sample sizes ranged from 18 to 34. Two studies had a duration of 4 weeks, and four studies lasted 8 weeks; only one study extended over a period of 12 months.

### 3.3. Effects of Interventions

#### 3.3.1. Energy Intake

**Figure 2:**
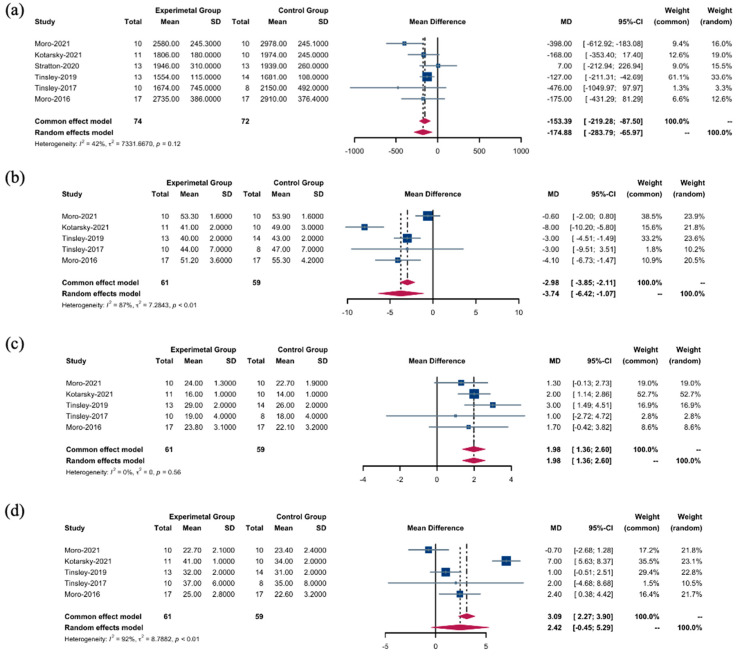
Energy intake between the TRF + RT and CON + RT interventions. (**a**) WMDs of overall energy intake between the TRF + RT and CON + RT interventions. (**b**) WMDs of energy intake of carbohydrates between the TRF + RT and CON + RT interventions. (**c**) WMDs of energy intake of proteins between the TRF + RT and CON + RT interventions. (**d**) WMDs of energy intake of fats between the TRF + RT and CON + RT interventions. In a meta-analysis, the “common effect model” (also known as the fixed-effect model) is utilized for low heterogeneity (*I*^2^ < 40%), presuming uniform effect sizes across studies, while the random-effects model is chosen for high heterogeneity (*I*^2^ > 40%) to account for varying effect sizes [18,22,25,26,27,28].

Using a random-effects model, we observed distinct nutritional effects across various intervention types; TRF + RT interventions were associated with a substantial reduction in overall energy intake compared to CON + RT interventions (WMD: −174.88 kcal; 95% confidence interval (CI): [−283.79; −65.97]; *p* = 0.0016). Despite the absence of significant heterogeneity, the moderately high *I*^2^ value of 42.3% (Figure 2a) indicated some degree of variance among the studies, although this was not sufficient to undermine the findings (Q (5) = 8.66, *p* = 0.1234).

This pattern was echoed in the consumption of carbohydrates, with TRF + RT interventions leading to a significant decrease in intake (WMD: −3.74 g; 95% CI: [−6.42; −1.07]; *p* = 0.0061). Here, heterogeneity was pronounced, as reflected by an *I*^2^ value of 87.4% (Figure 2b), suggesting a significant spread in effect sizes across studies (Q (4) = 31.70, *p* < 0.0001).

In contrast, when applying a fixed effects model to protein energy intake, the results supported the TRF + RT intervention as having a stronger effect (WMD: 1.98 g, 95% CI: [1.36 to 2.60]; *p* < 0.0001) (Figure 2c). Notably, no heterogeneity was detected (Q (4) = 2.96, *p* = 0.5643, *I*^2^ = 0.0%), suggesting a consistent effect across studies and strengthening the reliability of this finding.

However, fat intake did not differ significantly between the TRF + RT and CON + RT interventions according to the random effects model (WMD: 2.42 g; 95% CI: [−0.45, 5.29]; *p* = 0.0978). The effect sizes varied greatly (Q (4) = 53.18, *p* < 0.0001, *I*^2^ = 92.5%) (Figure 2d), indicating substantial heterogeneity. The variability highlights the complex dynamics at play and underscores the need for cautious interpretation of data in a given context.

#### 3.3.2. Body Composition

**Figure 3:**
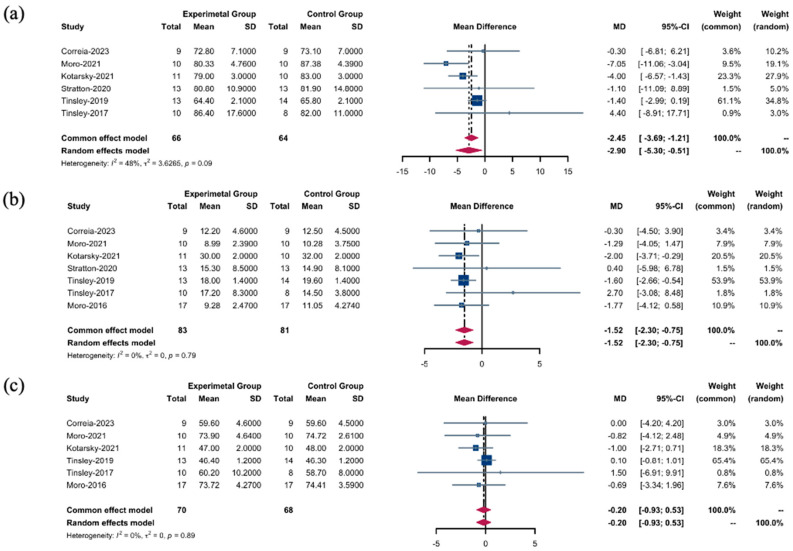
Body composition between the TRF + RT and CON + RT interventions. (**a**) WMDs of body mass between the TRF + RT and CON + RT interventions. (**b**) WMDs of fat mass between the TRF + RT and CON + RT interventions. (**c**) WMDs of fat-free mass between the TRF + RT and CON + RT interventions. In the meta-analysis, the “common effect model” (also known as the fixed-effect model) is utilized for low heterogeneity (*I*^2^ < 40%), presuming uniform effect sizes across studies, while the random-effects model is chosen for high heterogeneity (*I*^2^ > 40%) to account for varying effect sizes [18,22,24,25,26,27,28].

When evaluating the efficacy of the TRF + RT interventions, our analyses, stratified by effect models, yielded insightful findings regarding body composition outcomes. Using a random-effects model, we identified a notable decrease in BM in the TRF + RT group compared to the CON + RT group (WMD: −2.90 kg; 95% CI: [−5.30; −0.51]; *p* = 0.0175). Despite the lack of statistically significant disparities in heterogeneity, an *I*^2^ value of 48.1% (Figure 3a) indicated a mild level of variability among the studies, suggesting a spectrum of responses to the intervention (Q (5) = 9.64, *p* = 0.0862).

Further analysis using a fixed-effects model revealed a reduction in FM as a result of TRF + RT interventions (WMD: −1.52 kg; 95% CI: [−2.30, −0.75]; *p* = 0.0001). This result was characterized by the complete absence of heterogeneity (Q (6) = 3.11, *p* = 0.7945, *I*^2^ = 0.0%) (Figure 3b), indicating a consistent effect across different studies. However, when assessing changes in FFM, the fixed effects model revealed no significant differences between the TRF + RT and CON + RT groups (SMD: −0.20; 95% CI: [−0.93; 0.53]; *p* = 0.5941), corroborated by an *I*^2^ value of 0.0% (Figure 3c); this result underscores the uniformity of effect sizes across the studies (Q (5) = 1.69, *p* = 0.8899).

#### 3.3.3. Muscle Changes

**Figure 4:**
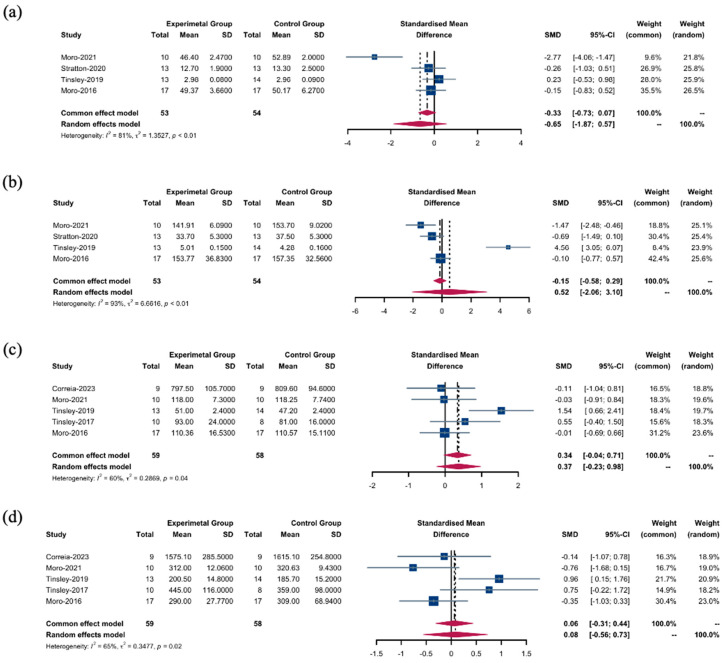
Muscles between the TRF + RT and CON + RT interventions. (**a**) SMDs of the cross-sectional area of upper limb muscles between the TRF + RT and CON + RT interventions. (**b**) SMDs of the cross-sectional area of lower limb muscles between the TRF + RT and CON + RT interventions. (**c**) SMDs of the muscle strength of upper limb muscles between the TRF + RT and CON + RT interventions. (**d**) SMDs of the muscle strength of lower limb muscles between the TRF + RT and CON + RT interventions. In the meta-analysis, the “common effect model” (also known as the fixed-effect model) is utilized for low heterogeneity (*I*^2^ < 40%), presuming uniform effect sizes across studies, while the random-effects model is chosen for high heterogeneity (*I*^2^ > 40%) to account for varying effect sizes [18,22,24,25,27,28].

Employing a random-effects model, we investigated the differences in muscle cross-sectional area and strength in response to the TRF + RT and CON + RT interventions. These interventions did not lead to significant differences in the cross-sectional areas of the upper (SMD: −0.65; 95% CI: [−1.87; 0.57]; *p* = 0.2966) (Figure 4a) or lower limb muscles (SMD: 0.52; 95% CI: [−2.06, 3.10]; *p* = 0.6928) (Figure 4b). Similarly, comparisons of muscle strength of the upper (SMD: 0.37; 95% CI: [−0.23; 0.98]; *p* = 0.2297) (Figure 4c) and lower limbs (SMD: 0.08; 95% CI: [−0.56; 0.73]; *p* = 0.7990) (Figure 4d) revealed no significant changes.

#### 3.3.4. Biomarkers

**Figure 5:**
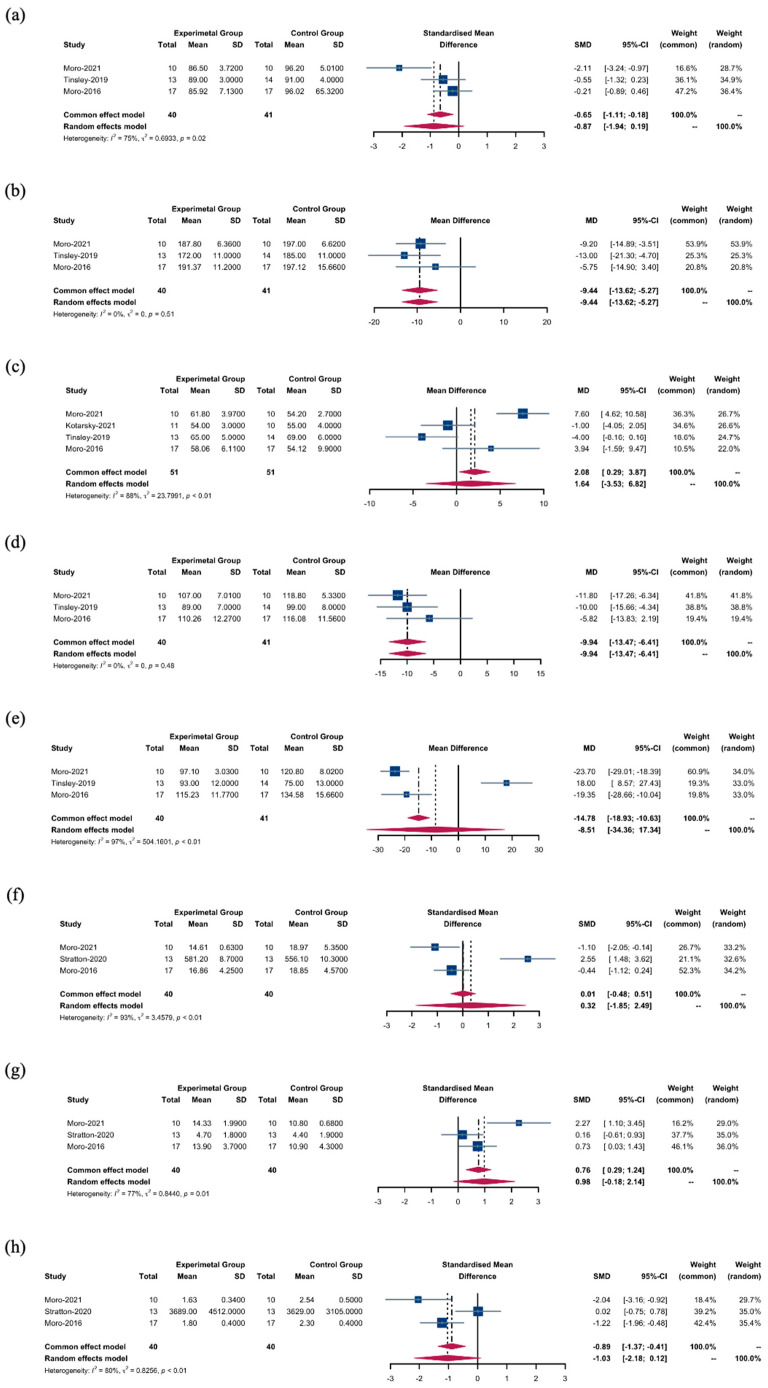
Biomarkers between the TRF + RT and CON + RT interventions. (**a**) SMDs of blood glucose levels between the TRF + RT and CON + RT interventions. (**b**) WMDs of total cholesterol levels between the TRF + RT and CON + RT interventions. (**c**) WMDs of HDL-C levels between the TRF + RT and CON + RT interventions. (**d**) WMDs of LDL-C levels between the TRF + RT and CON + RT interventions. (**e**) WMDs of triglyceride levels between the TRF + RT and CON + RT interventions. (**f**) SMDs of testosterone levels between the TRF + RT and CON + RT interventions. (**g**) SMDs of adiponectin levels between the TRF + RT and CON + RT interventions. (**h**) SMDs of leptin levels between the TRF + RT and CON + RT interventions. In the meta-analysis, the “common effect model” (also known as the fixed-effect model) is utilized for low heterogeneity (*I*^2^ < 40%), presuming uniform effect sizes across studies, while the random-effects model is chosen for high heterogeneity (*I*^2^ > 40%) to account for varying effect sizes [18,22,25,26,27].

We investigated the effects of TRF + RT versus CON + RT on several biomarkers using random- and fixed-effects models. Initial findings indicated that blood glucose levels were not significantly altered by TRF + RT interventions (SMD: −0.87; 95% CI: [−1.94; 0.19]; *p* = 0.1088), despite notable heterogeneity among the studies (*I*^2^ = 75.0%) (Figure 5a). A more consistent pattern emerged with total cholesterol, where it was significantly reduced in the TRF + RT group (WMD: −9.44 mg/dL; 95% CI: [−13.62; −5.27]; *p* = 0.0001), with the studies showing uniformity in effect sizes (*I*^2^ = 0.0%) (Figure 5b).

In contrast, our analysis found no significant difference in high-density lipoprotein cholesterol (HDL-C) between the intervention groups (WMD: 1.64 mg/dL; 95% CI: [−3.53; 6.82]). However, the high statistical heterogeneity (*I*^2^ = 88.4%) (Figure 5c) indicates that factors beyond the interventions may influence HDL-C levels. In addition, low-density lipoprotein cholesterol (LDL-C) levels were significantly decreased in the TRF + RT group (WMD: −9.94 mg/dL; 95% CI: [−13.47; −6.41]; *p* = 0.0001) without heterogeneity (*I*^2^ = 0.0%) (Figure 5d); insulin levels were also significantly reduced (SMD: −0.72; 95% CI: [−1.24; −0.21]; *p* = 0.0059), albeit with moderate heterogeneity (*I*^2^ = 41.4%). Conversely, the random effects model showed no significant difference in triglyceride levels between the TRF + RT and CON + RT groups (WMD: −8.51 mg/dL; 95% CI: [−34.36, 17.34]; *p* = 0.5187) (Figure 5e).

No significant differences in testosterone levels were observed between the groups (SMD: 0.32; 95% CI: [−1.85; 2.49]; *p* = 0.7745) (Figure 5f). Adiponectin levels also remained unchanged (SMD: 0.98; 95% CI: [−0.18; 2.14]; *p* = 0.0980) (Figure 5g). Finally, leptin levels did not differ significantly between the TRF + RT and CON + RT groups (SMD: −1.03; 95% CI: [−2.18; 0.12]; *p* = 0.0779) (Figure 5h).

### 3.4. Risk of Bias

**Figure 6:**
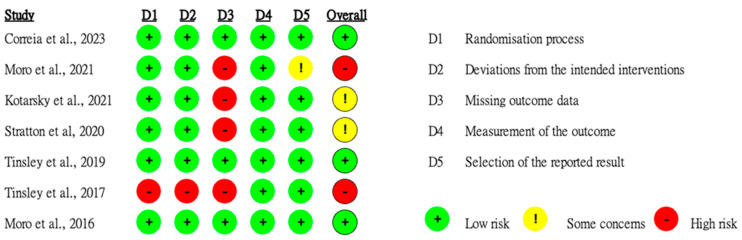
Quality assessment of the selected studies [18,22,24,25,26,27,28].

Of the seven studies analyzed, three were classified as low risk of bias, two as some concerns, and two as high risk. One study did not provide sufficient information regarding the randomization process and showed significant differences between the experimental and control groups at baseline. Some studies reported that participants were blinded to the intervention. A slight possibility was detected with regard to participants or researchers influencing the measurements and outcomes. However, four studies appeared to have a high risk of bias owing to missing outcome data (Figure 6).

### 3.5. Sensitivity Analyses and Publication Bias

A series of sensitivity analyses were performed by removing each study individually, with no considerable changes to the results. Daily energy intake, BM, FM, and FFM were each represented by a minimum of six studies, allowing for an assessment of publication bias for these variables. The resulting funnel plot exhibited approximate symmetry, and Egger’s test showed no indication of publication bias for daily energy intake (*p* = 0.38), BM (*p* = 0.929), FM (*p* = 0.05), and FFM (*p* = 0.595). Consequently, the potential for publication bias in these analyses was deemed to be low (Supplementary Materials Figures S1–S4). However, it is conventionally advised to have a minimum of 10 studies to generate a funnel plot. Given the limited number of studies, these findings should be treated with caution.

## 4. Discussion

The key finding of the present study was that the combined TRF + RT approach resulted in effective weight and fat loss. The significant reductions in body weight (−2.9 kg), FM (−1.52 kg), insulin levels, total cholesterol, and LDL-C, as observed in this analysis, indicate the efficacy of TRF + RT compared to CON + RT. In particular, as the TRF + RT combination did not significantly reduce muscle mass or strength, it has a unique potential for preserving FFM, which is a common challenge in conventional weight loss methods [29]. Therefore, TRF, when paired with RT, may offer a balanced approach to weight management by targeting fat loss while maintaining muscle mass [18]. These findings are consistent with previous meta-analyses that showed a significant difference in body weight and FM while preserving FFM between TRF or intermittent feeding + exercise and control diet + exercise groups [20,30,31]. 

In terms of the TRF strategy, the majority of studies adhered to a 16:8 protocol, designating an 8 h eating window typically ranging from 12 pm to 8 pm or 1 pm to 9 pm. This protocol’s consistency reflects a high degree of uniformity in TRF application across the majority of the studies, with adherence to the 16:8 schedule being notably good. However, the less conventional 4 h eating window, as explored in one particular study [28], was associated with a high participant dropout rate. This shorter window regimen was perceived as more difficult to adhere to by some subjects, and it yielded considerable variability in outcomes among individuals. Notably, the energy intake in the TRF + RT group was 174 kcal lower than that in the CON + RT group, although the TRF intervention did not require participants to restrict energy intake. This reduction aligns with that reported in a previous review, where intermittent fasting combined with exercise led to a 140 kcal/day decrease in energy intake [30], suggesting that the observed weight loss could be a consequence of an accidental caloric deficit caused by TRF [32,33]. This reduction in energy intake without deliberate calorie counting underscores the potential utility of TRF in weight management and could be a pivotal aspect of its success. Further research is required to elucidate how TRF leads to reduced energy intake and its relationship with weight loss.

In our review, we categorized the included studies based on the duration of the TRF + RT intervention: two studies spanned 4 weeks, four extended to 8 weeks, and one lasted a full 12 months. Among them, the 4-week interventions showed no significant effects. In contrast, three out of the four 8-week interventions led to a decrease in weight ranging from 1.4 to 4 kg and body fat from 1.6 to 2 kg. Notably, the 12-month study demonstrated a substantial reduction in weight and body fat of 7 kg and 2.9 kg, respectively. From these findings, we infer that an 8-week period may represent the shortest effective intervention duration for TRF + RT, with the possibility of enhanced benefits with extended durations. The finding is also supported by a previous systematic review [20]. Nevertheless, the current evidence is sparse, and further research is needed to delineate the optimal duration for intervention.

However, the effects showed variability and were not consistently significant across studies. Its impact on blood lipid levels remained inconclusive. While some research consistently indicates that TRF may help lower LDL-C levels [20,30,34], the impact on total cholesterol, triglyceride, and HDL-C remains inconclusive [20,30,34,35,36]. Our meta-analysis observed modest improvements in cholesterol levels, which could be linked to a reduction in body fat, decreased caloric intake, and dietary habit alterations. TRF likely lowers blood lipids by reducing liver enzymes like fatty acid synthase and peroxisome proliferator-activated receptor gamma, altering lipid metabolism and promoting fat oxidation [37,38]. Additionally, TRF improves insulin sensitivity and reduces liver gluconeogenesis, further contributing to lower triglyceride and cholesterol levels [37]. However, the specific biological mechanisms underlying these changes necessitate further investigation.

The review revealed marked improvements in insulin levels, yet no significant changes were noted in glucose, triglycerides, and adipose tissue hormones, which are typically linked to insulin resistance. Many previous human studies [10,39,40], as well as animal studies [41], have shown that TRF improved insulin sensitivity. However, the nonsignificant findings regarding glucose profiles are consistent with some studies [20,35] and inconsistent with some other studies, which showed that TRF has the potential to improve metabolic markers, such as blood glucose and leptin [10,20,30]. TRF enhances insulin sensitivity and lowers blood glucose levels by aligning energy intake with circadian rhythms, promoting metabolic shifts, and reducing oxidative stress and inflammation [10,40,41]. Additionally, it modulates fat oxidation and clock gene expression, further improving metabolic health [39,41]. Improvements in insulin sensitivity could precede or occur independently from shifts in triglyceride levels or adipose tissue hormone secretion. Our results indicate a positive trend in adiponectin and leptin profiles, yet the meta-analysis was based on only three studies with existing heterogeneity. This limited and varied evidence may explain the nonsignificant outcomes, underscoring the need for additional research to investigate these effects further.

The variation suggests that the effectiveness of TRF + RT in improving these particular outcomes may depend on individual factors, such as participant baseline health status, specific training regimens, or adherence to the TRF protocol. Therefore, whereas TRF + RT shows promise for weight and dietary management, its effect on muscle development, strength enhancement, and some aspects of metabolic health needs to be conclusively determined as it may be influenced by personal and program-related variables.

This study had some limitations, including the small number of included studies with associated potential bias risks, indicating the existence of challenges with regard to the generalization of the results. The observed heterogeneity and individual differences underscore the substantial influence of baseline health status, specific training programs, and adherence to TRF protocols on outcomes, as well as that of study design, highlighting the need for further research on individualized factors. Additionally, inconsistent results regarding metabolic markers such as blood glucose, triglyceride, leptin, adiponectin, and testosterone levels suggest the need for more comprehensive assessments to fully understand their effects on metabolic health, highlighting areas for future exploration. Our findings highlight the complexity of metabolic and hormonal responses and the importance of personalized intervention approaches. In addition, none of the studies included have monitored the persistence of effects after an intervention, indicating a notable lack of longitudinal data. Future research endeavors should consider incorporating long-term follow-up to assess the enduring impacts of interventions.

## 5. Conclusions

Our study explored the synergistic effects of TRF combined with RT on adult body composition and metabolic health, providing a comprehensive approach for health optimization beyond traditional diet or exercise interventions alone. Our findings highlight the significant health benefits, including improved body composition, reduced insulin, total cholesterol, and LDL-C levels, and decreased dietary intake, showcasing the efficacy of TRF + RT compared with that of CON + RT in promoting weight and FM reduction while potentially preserving lean mass and strength, offering a new perspective on balanced weight management.

## Figures and Tables

**Table 1:** Characteristics of the trials included in the meta-analysis.

Author, Year	Participants	Study Design	Duration	TRF Intervention	RT Intervention	Energy Intake (kcal)	Results
Moro et al. (2016) [22]	*n* = 34, trained males, TRF + RT (*n* = 17): age = 29.9 ± 4.07 y BM = 83.9 ± 12.8 kg CON + RT (*n* = 17): age = 28.5 ± 3.48 y BM = 85.3 ± 13 kg	RCT	8 weeks	TRF: 8 h eating window, 1:00–8:00 p.m. Ad libitum CON: habitual dietary patterns 20 g of whey protein after training for both groups	6–8 repetitions at 85–90% 1RM, 3 times/week	TRF: 2735 ± 386 CON: 2910 ± 376.4	TRF + RT vs. CON + RT: BM ↓, FM ↓, adiponectin ↑, leptin ↓, IL-1β ↓, testosterone ↓, IGF-1 ↓, TG ↓, IL-6 ↓, TNF-α ↓ TRF + RT: insulin ↓, glucose ↓, HDL-C ↑ TRF + RT and CON + RT: FFM ↔, arm and thigh muscle CSA ↔, leg and bench press 1RM ↔
Correia et al. (2023) [24]	*n* = 18, healthy and trained males, age = 23.7 ± 2.6 y, TRF + RT (*n* = 9): BM = 73.2 ± 7.2 kg CON + RT (*n* = 9): BM = 73.0 ± 6.9 kg	crossover RCT	4 weeks	TRF: 8 h eating window, 1:00–9:00 p.m. Ad libitum CON: habitual dietary patterns	4 sets of maximum repetitions at 85% 1RM in 5 exercises, 3 times/week	TRF: 2433.3 ± 760.5 CON: 2427.0 ± 556.8	TRF + RT vs. CON + RT: lower body muscle strength ↓; upper body muscle strength ↑ TRF + RT and CON + RT: FM ↓, BM ↔, FFM ↔
Moro et al. (2021) [25]	*n* = 20, trained males, TRF + RT (*n* = 10): BMI = 26.5 kg/m^2^ CON + RT (*n* = 10): BMI = 27.2 kg/m^2^	RCT	12 months	TRF: 8 h eating window, 1:00–8:00 p.m. Ad libitum CON: habitual dietary patterns 20 g of whey protein after training for both groups	6–8 repetitions at 85–90% 1RM, 3 times/week	TRF: 2580.0 ± 245.3 CON: 2978.0 ± 245.1 *	TRF + RT vs. CON + RT: BM ↓, FM ↓, FFM ↓, arm and thigh muscle CSA ↓, TG ↓, HDL ↑, inflammatory markers (IL-6 ↓, IL-1β ↓, TNF-α ↓) insulin sensitivity (fasting glucose ↓, insulin ↓, HOMA-IR ↓), IGF-1 ↓, testosterone ↓, adiponectin ↑, leptin ↓ TRF + RT and CON + RT: leg press 1RM ↑, bench press 1RM ↑
Kotarsky et al. (2021) [26]	*n* = 21 (M/3) overweight and obese adults TRF + RT (*n* = 11): age= 45 ± 3 y BMI = 29.8 ± 0.8 kg/m^2^ CON + RT (*n* = 10): age = 44 ± 2 y BMI = 29.4 ± 0.8 kg/m^2^	RCT	8 weeks	TRF: 8 h eating window (12:00–8:00 p.m. Ad libitum) CON: habitual dietary patterns	3 resistance training routines, 3 sets of 12 repetitions, 3–4 times/week	TRF: 1806 ± 180 (306 kcal less) CON: 1974 ± 245 (253 kcal less)	TRF + RT vs. CON + RT: BM ↓, FM ↓,VFM ↓, knee extension strength ↓, TRF + RT and CON + RT: BMI ↓,WC ↓, FFM ↑, knee flexion strength ↑, dorsiflexion strength ↑, cardiometabolic biomarkers ↔ (insulin, hsCRP, HbA1c, TC, and HDL) hormone levels ↔ (estradiol, progesterone, testosterone, cortisol)
Stratton et al. (2020) [27]	*n* = 26, recreationally active and trained males, TRF + RT (*n* = 13): age = 22.9 ± 3.6 y BM = 82.0 ± 10.6 kg CON + RT (*n* = 13): age = 22.5 ± 2.2 y BM = 83.3 ± 15.0 kg	RCT	4 weeks	TRF: 8 h eating window (12:00–8:00 p.m., or 1:00–9:00 p.m.) both groups (TRF and CON): 25% caloric deficit	2–4 sets of 4–15 repetitions at 70–87.5% 1RM, 3 times/week	TRF: 1946 ± 310 CON: 1939 ± 260	TRF + RT vs. CON + RT: FM ↔, cortisol ↓ TRF + RT and CON + RT: BM ↓, FM ↓, FFM ↔, leg press 1RM ↑, bench press 1RM ↑, VL CSA ↑, BB CSA ↑, testosterone ↓, adiponectin ↓, leptin ↓, ghrelin ↓
Tinsley et al. (2019) [18]	*n* = 40, trained females, TRF + RT (*n* = 13): age = 23.3 ± 1.5 y BMI = 23.8 kg/m^2^ CON + RT (*n* = 14): age = 22.6 ± 2.7 y BMI = 22.5 kg/m^2^	RCT	8 weeks	TRF: 8 h eating window (12:00–8:00 p.m.) both groups (TRF and CON): ≥1.4 g/kg/d protein intake; energy intake per day = REE * 1.5–250 kcal	alternating 2 different upper and lower body exercise, 3 times/week	TRF: 1624 ± 107 CON: 1570 ± 111	TRF + RT vs. CON + RT: FM ↓ TRF + RT and CON + RT: BM ↑, FFM ↑, muscle thickness ↑, leg press 1RM ↑, bench press 1RM ↑
Tinsley et al. (2017) [28]	*n* = 28, recreationally active males, TRF + RT (*n* = 14): age = 22.9 ± 4.0 y BM = 87.4 ± 19.2 kg CON + RT (*n* = 14): age = 22.0 ± 2.4 y BM = 79.0 ± 13.5 kg	RCT	8 weeks	TRF: 4 days/week (non-training days), 4 h eating window (any 4 h window between 4:00 p.m. and midnight) 3 days/week (training days): ad libitum CON: habitual dietary patterns	alternating upper and lower body exercise, 4 sets of 8–12 repetitions, 3 times/week	TRF: 1878 ± 532 CON: 2107 ± 694 *	TRF + RT and CON + RT: BM ↔, LBM ↔, FM ↔, hip sled endurance ↑, hip sled 1RM ↑, bench press 1RM ↑

Values are presented as mean ± standard deviation (SD). One RM: one repetition maximum; BB: biceps brachii; BMI: body mass index; BM: body mass; CSA: cross-sectional area; FFM: fat-free mass; IGF-1: insulin-like growth factor 1; IL-1β: interleukin-1 beta; M: male; RCT: randomized controlled trial; REE: resting energy expenditure; RT: resistance training; TRF: time-restricted feeding; TRF + RT: time-restricted feeding combined with resistance training; CON + RT: habitual meal pattern combined with resistance training; VFM: visceral fat mass; WC: waist circumference; y: years. ↑ indicates increased; ↓ indicates reduced; ↔ indicates no statistically significant difference between measures; vs. denotes comparison with the following condition. * *p* < 0.05.

## Supplementary Materials

The following supporting information can be downloaded at: https://www.mdpi.com/article/10.3390/nu16183066/s1, Figure S1: Funnel plot of comparison between TRF + RT and CON + RT on daily energy intake; Figure S2: Funnel plot of comparison between TRF + RT and CON + RT on body mass; Figure S3: Funnel plot of comparison between TRF + RT and CON + RT on fat mass; Figure S4: Funnel plot of comparison between TRF + RT and CON + RT on fat free mass.
