# Supplementary material for: Synergistic Effects of Time-Restricted Feeding and Resistance Training on Body Composition and Metabolic Health: A Systematic Review and Meta-Analysis
Source: Nutrients. 2024 Sep 11;16(18):3066. doi: 10.3390/nu16183066 (PMC11434652; doi:10.3390/nu16183066)
Supplement: Supplementary file 1 [file nutrients-16-03066-s001.zip › nutrients-3158197-supplementary.pdf]

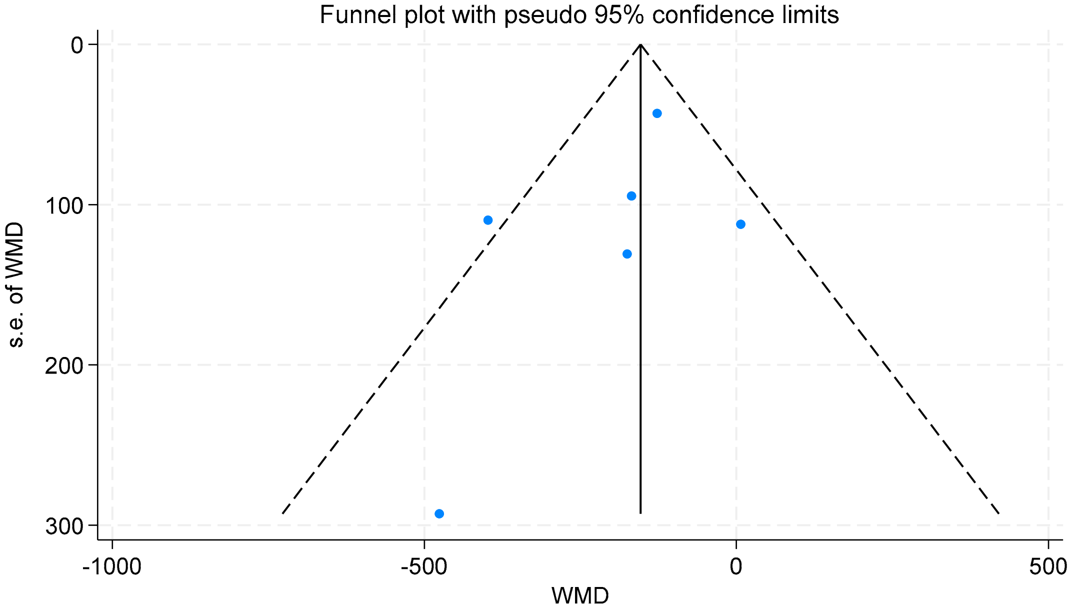

Egger’s test P = 0.380

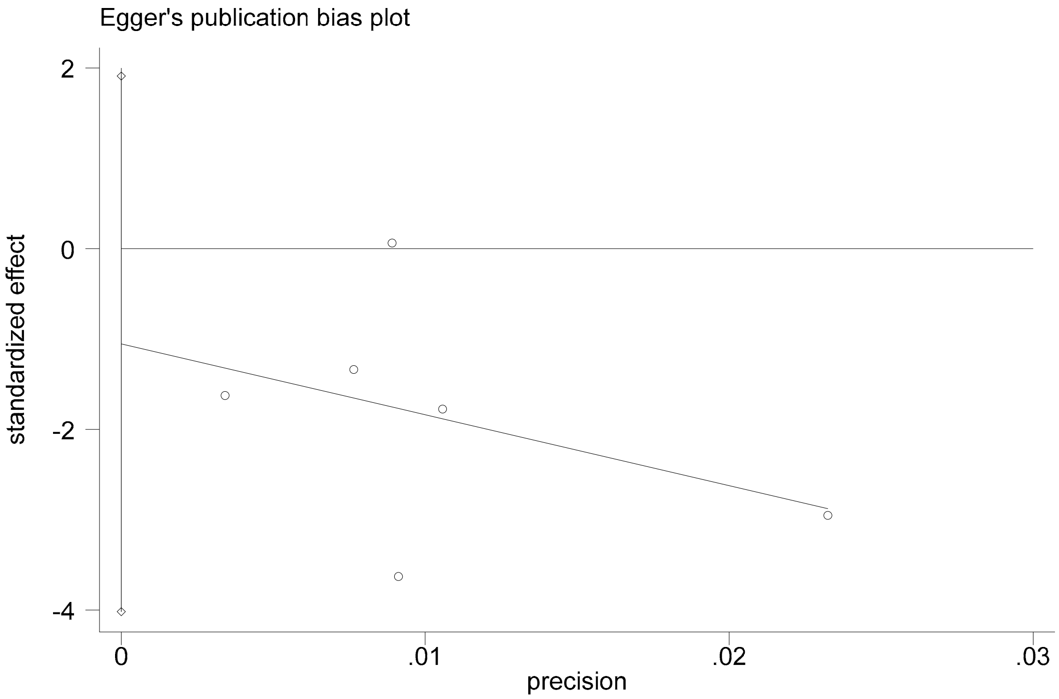

Figure S1. Funnel plot of comparison between TRF+RT and CON+RT on daily energy intake.

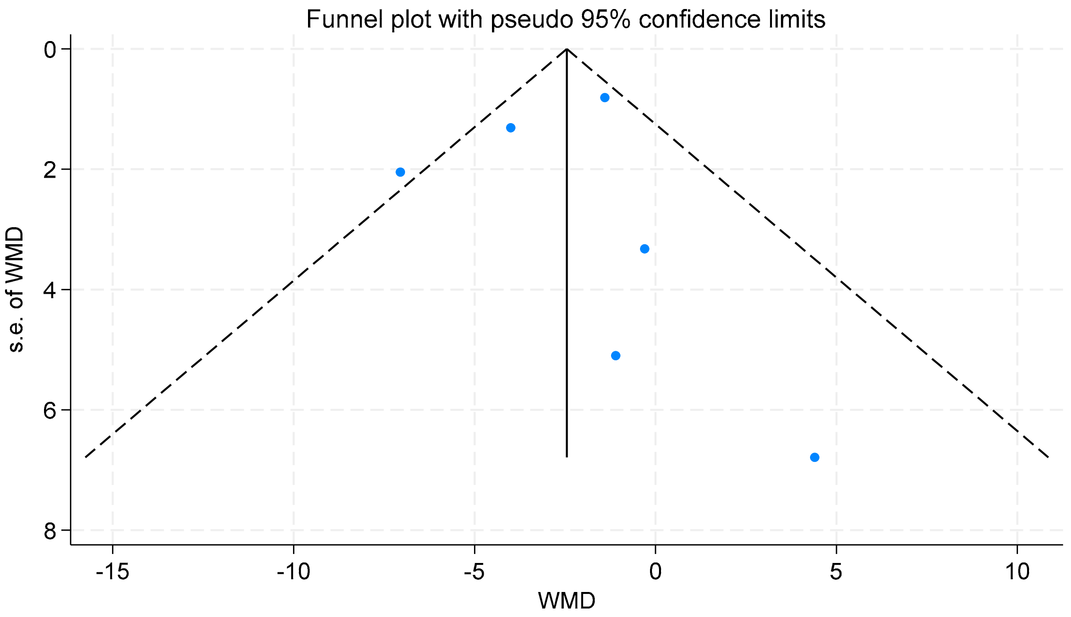

Egger's test P = 0.929

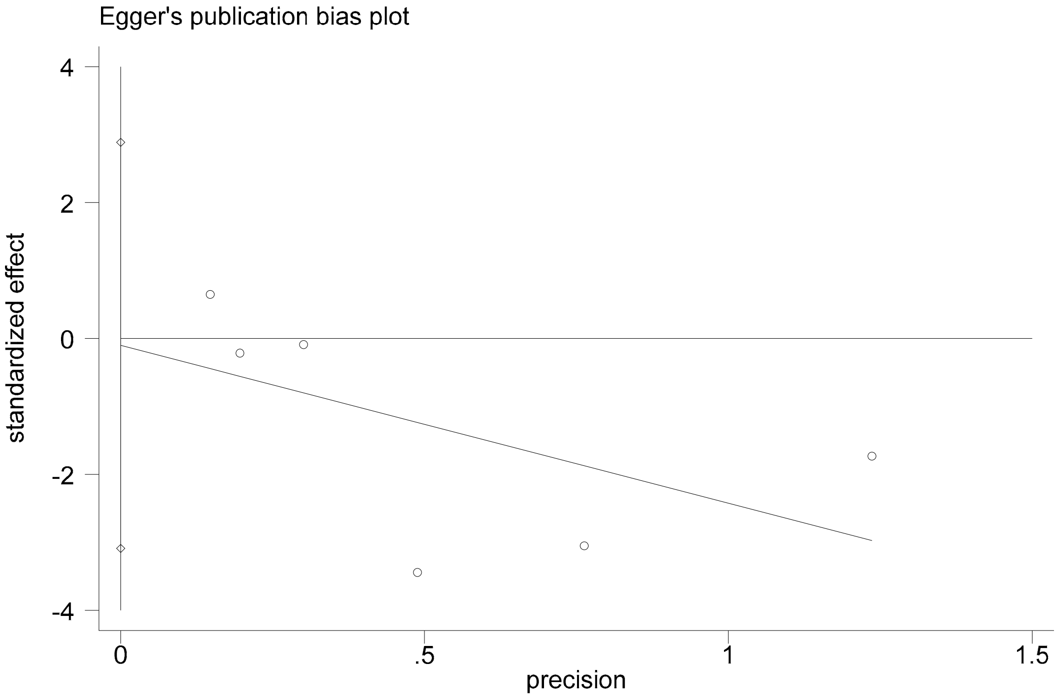

Figure S2. Funnel plot of comparison between TRF+RT and CON+RT on body mass.

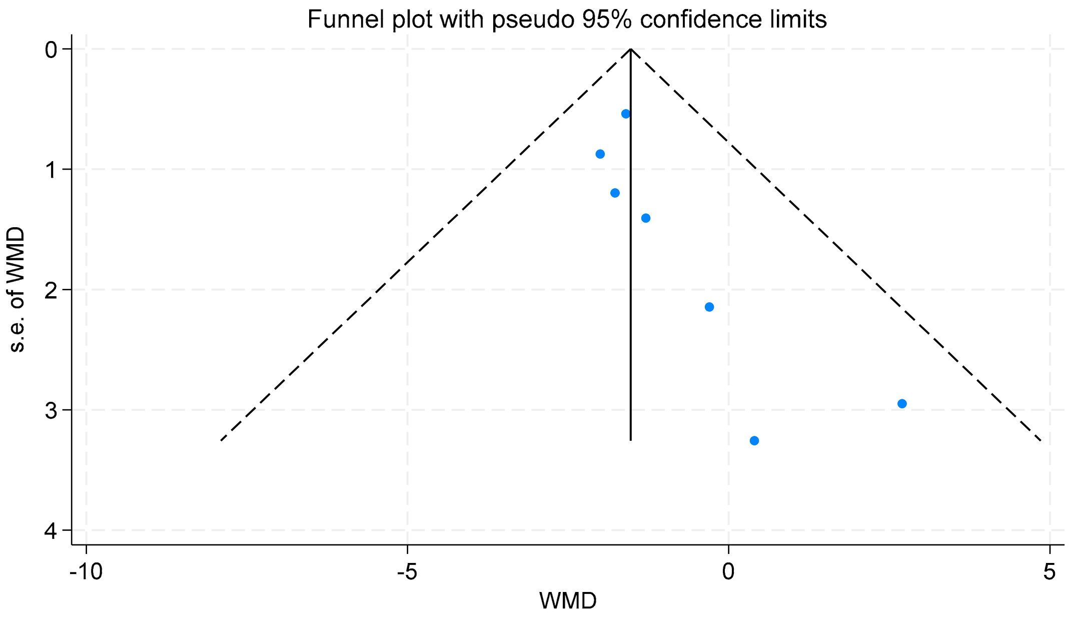

Egger’s test P = 0.050

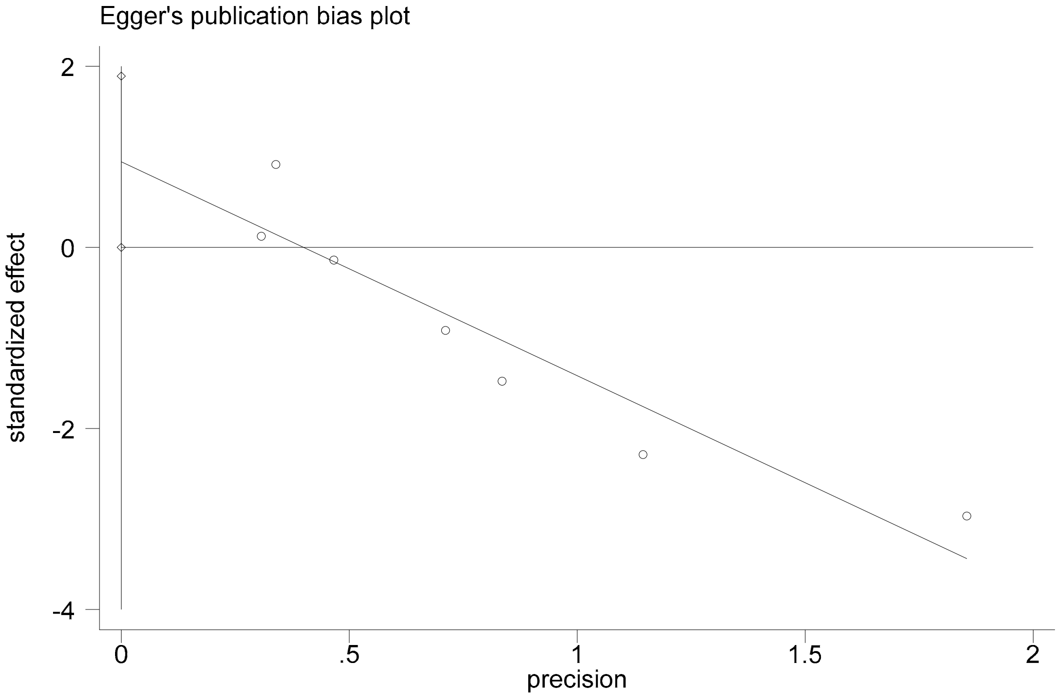

Figure S3. Funnel plot of comparison between TRF+RT and CON+RT on fat mass.

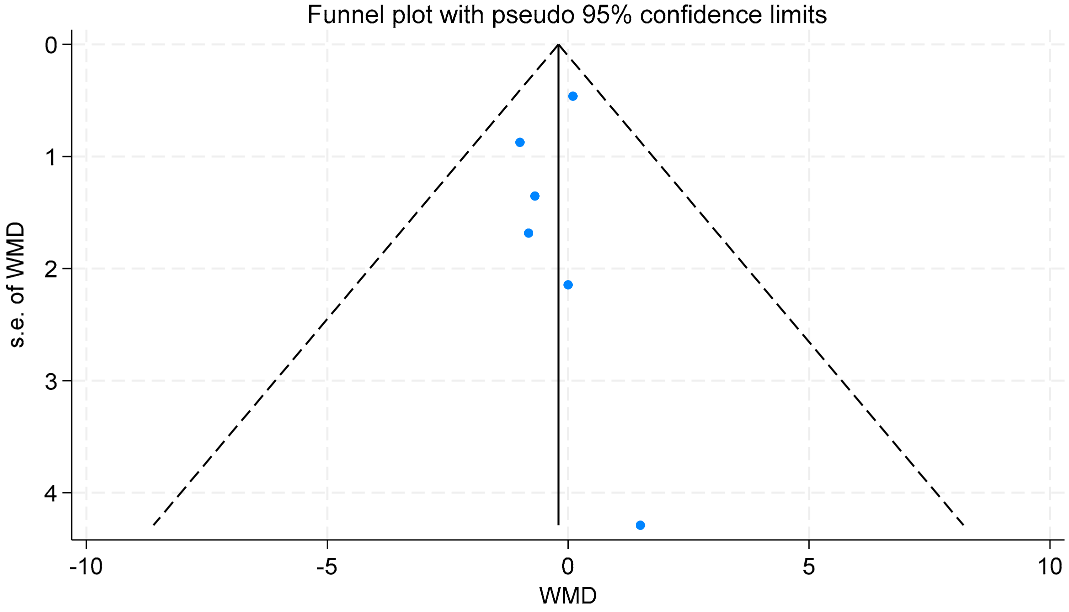

Egger’s test P = 0.595

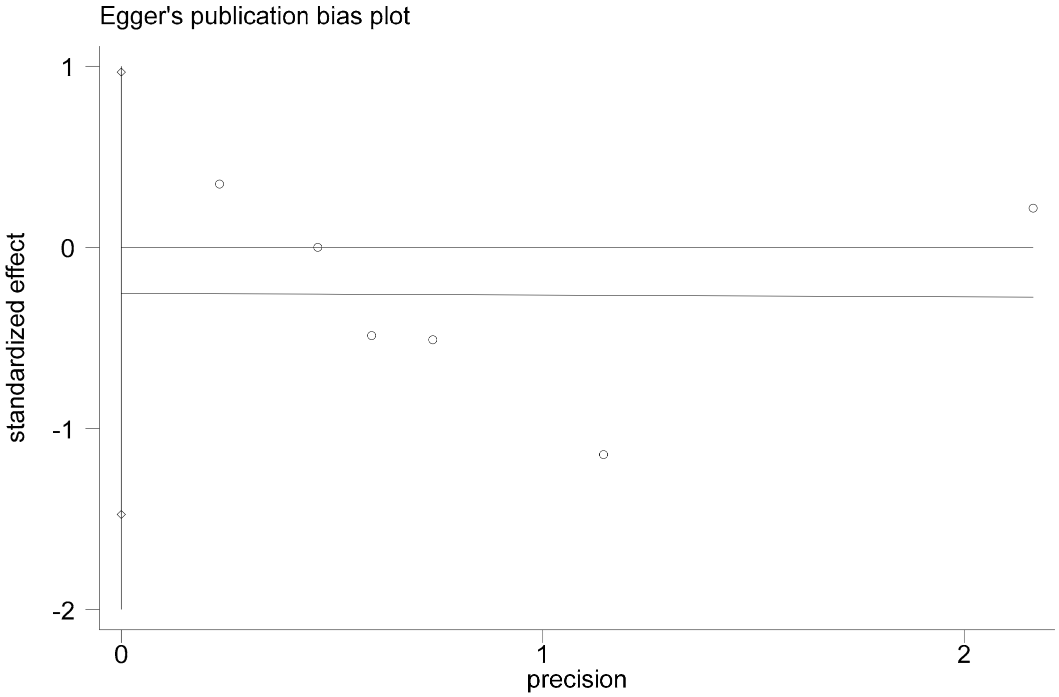

Figure S4. Funnel plot of comparison between TRF+RT and CON+RT on fat free mass.
